# Supplementary material for: Does age matter?—Efficiency of mechanical food break down in Tupaia belangeri at different ages
Source: PLoS One. 2023 Jul 10;18(7):e0274439. doi: 10.1371/journal.pone.0274439 (PMC10411959; doi:10.1371/journal.pone.0274439)
Supplement: S1 Table — (DOCX) [file pone.0274439.s001.docx]

S1 Table: Model coefficients from a linear mixed effect model for the sample size by age class.

| **term** | **estimate** | **CI** | **p-value** |
| --- | --- | --- | --- |
| (Intercept) | 13869 | [ 9286; 18452] | < 0.01 |
| age (adult) | -5658 | [-11993; 676] | 0.08 |
| age (senile) | -10728 | [-16504; -4951] | < 0.01 |
