# Supplementary material for: Does age matter?—Efficiency of mechanical food break down in Tupaia belangeri at different ages
Source: PLoS One. 2023 Jul 10;18(7):e0274439. doi: 10.1371/journal.pone.0274439 (PMC10411959; doi:10.1371/journal.pone.0274439)
Supplement: S2 Table — (DOCX) [file pone.0274439.s002.docx]

S2 Table: Model coefficients from a linear mixed effect model for the particle size by age class controlled for number of particles per sample in the interaction.

| **term** | **estimate** | **95% CI** | **p value** |
| --- | --- | --- | --- |
| (Intercept) | 1.25 | [-1.37; 3.88] | 0.355 |
| age (adult) | 2.48 | [-1.16; 6.11] | 0.188 |
| age (senile) | -0.95 | [-3.96; 2.06] | 0.539 |
| sample size | -0.36 | [-0.64; -0.08] | 0.016 |
| age (adult):sample size | -0.29 | [-0.69; 0.12] | 0.171 |
| age (senile):sample size | 0.08 | [-0.26; 0.43] | 0.638 |
