# Supplementary material for: Does age matter?—Efficiency of mechanical food break down in Tupaia belangeri at different ages
Source: PLoS One. 2023 Jul 10;18(7):e0274439. doi: 10.1371/journal.pone.0274439 (PMC10411959; doi:10.1371/journal.pone.0274439)
Supplement: S3 Table — (DOCX) [file pone.0274439.s003.docx]

S3 Table: Model coefficients from a mixed effect logistic regression model for extremely large particle size by age class controlled for number of particles per sample in the interaction.

| **term** | **level** | **term2** | **estimate** | **CI** | **p.value** |
| --- | --- | --- | --- | --- | --- |
| (Intercept) |  |  | 0.003 | [0.002; 0.005] | < 0.001 |
| age | juvenile |  |  |  |  |
| age | adult |  | 1,982 | [1.040; 3.776] | 0.038 |
| age | senile |  | 12,163 | [6.352; 23.291] | < 0.001 |
| sample size |  |  | 0.594 | [0.480; 0.736] | < 0.001 |
| age | juvenile | * |  |  |  |
| age | adult | ProbeSize | 0.494 | [0.333; 0.733] | < 0.001 |
| age | senile | ProbeSize | 0.369 | [0.231; 0.589] | < 0.001 |
