# Supplementary material for: Does age matter?—Efficiency of mechanical food break down in Tupaia belangeri at different ages
Source: PLoS One. 2023 Jul 10;18(7):e0274439. doi: 10.1371/journal.pone.0274439 (PMC10411959; doi:10.1371/journal.pone.0274439)
Supplement: S4 Table — (DOCX) [file pone.0274439.s004.docx]

S4 Table: Model coefficients from a mixed effect logistic regression model for extremely small particle size by age class controlled for number of particles per sample in the interaction.

| **term** | **level** | **term2** | **estimate** | **CI** | **p.value** |
| --- | --- | --- | --- | --- | --- |
| (Intercept) |  |  | 0.010 | [0.008; 0.013] | < 0.001 |
| age | juvenile |  |  |  |  |
| age | adult |  | 0.206 | [0.139; 0.305] | < 0.001 |
| age | senile |  | 0.553 | [0.340; 0.899] | 0.017 |
| sample size |  |  | 1,315 | [1.081; 1.599] | 0.006 |
| age | juvenile | * |  |  |  |
| age | adult | ProbeSize | 1,037 | [0.719; 1.497] | 0.845 |
| age | senile | ProbeSize | 0.689 | [0.397; 1.196] | 0.186 |
